# Building and Maintaining Self-Efficacy Beliefs: A Study of Entry-Level Vocational Education and Training Teachers

**DOI:** 10.1007/s12186-023-09326-x

**Published:** 2023-07-17

**Authors:** Nathalie Gagnon, Annie Dubeau

**Affiliations:** 1 Université du Québec à Rimouski, Rimouski, Québec Canada; 2 Université du Québec à Montréal, Montréal, Québec Canada

**Keywords:** New teachers, Professional induction, Vocational training, Vocational education, Perceived self-efficacy

## Abstract

New vocational education and training (VET) teachers in Quebec (Canada), as in other countries (e.g., France, the Netherlands, Norway, Sweden, Switzerland, the United States), face specific challenges and experience an atypical process of entry into the teaching profession. In addition to the known professional induction challenges, which requires numerous adjustments in terms of organizational integration and socialization as well as on a personal level, new VET teachers also experience a major professional and identity transition: they shift from experts in their field to novices in the teaching field. Moreover, as they are not generally trained in pedagogy, new teachers must enroll in a mandatory teaching bachelor’s program once they are fully in charge of a class and its educational responsibilities. This complex process calls for a closer look at ways to facilitate their induction experience. Specifically, because it is closely related to motivation, engagement, and performance, this study focuses on their perceived self-efficacy as teachers. This study focused on 21 new VET teachers in the francophone province of Quebec, Canada, and aim to identify different strategies through which they succeeded in developing and maintaining their self-efficacy: strategies related to 1) the work of teaching, 2) mobilization of resources, 3) professional development, and 4) attitudes and well-being at work. These strategies allow us to gain a deeper understanding of previously unexplored aspects of the reality of these teachers, and to propose avenues for the development of interventions targeting their needs.

## Introduction: Challenges related to the induction process of VET teachers

It is known that the teachers’ induction process comes with significant challenges (Colognesi et al., 2020; Gagnon, 2017; Mukamurera et al., 2019). However, in Canada, few studies have focused specifically on the early career in vocational education. Yet, the backgrounds and contexts in which vocational education and training (VET) teachers work differ greatly from elementary and high school. Indeed, in addition to this induction process, VET teachers are going through an important transition in their professional identity, shifting from being experts in their trade to novices in pedagogy (Balleux et al., 2016; Beaucher, 2021; Berger et al., 2018; Boldrini et al., 2018; Coppe et al., 2021; Deever et al., 2020; Fejes & Köpsén, 2014; Geeraerts et al., 2015; Perez-Roux, 2010; Sarastuen, 2020; Tigchelaar et al., 2008). While they take part in a process of knowledge and skill construction, work socialization, and identity transformation (Martineau et al., 2008), this induction process also leads them toward “the discovery of new values […], guides the construction or reconstruction of professional identities, representations of the profession, and expectations of corresponding roles” (Dupuy & Leblanc, 2001, p. 70). In order to consider the special status of these teachers who enter the profession with a wealth of experience and knowledge, some authors rather refer to the concept of work socialization (Martineau et al., 2009). Induction is then conceived as a bidirectional process, where the new teachers are involved in an ongoing learning process (about the new professional role), and outgoing recognition process, recognized as a source of expertise for the work environment (Coppe et al., 2023).

Before presenting the challenges associate with this process, the following section exposes the context of this study, with a description of Quebec’s unique vocational education system.

## Research Context: Vocational Education and Training Programs in Quebec, Canada

The issues related to VET in the Canadian francophone province of Quebec (8,700,000 inhabitants) are similar to those in several other countries (e.g., France, the Netherlands, Norway, Sweden, Switzerland, the United States). In this province, secondary-level vocational training programs^1^ are designed to provide training for a skilled or semi-skilled trade (Gouvernement du Québec, 2021). Through more than 190 educational institutions located throughout the province of Quebec (Gouvernement du Québec, 2021), 147 programs (e.g., retail butchery, bricklaying, hairdressing, horticultural production, etc.) are offered divided in 21 major sectors (e.g., Administration, commerce and computer technology, Food services and tourism, Arts, etc.). These programs vary in length from 600 to 1,800 h. In 2020–2021, vocational training programs in the Quebec public education network enrolled over 120,000 students, taught by approximately 10,000 teachers (Institut de la statistique du Québec, 2022).

Most of these VET teachers are recruited by Vocational training centers^2^ (VTC) based on their professional experience in a professional trade. They are hired without having received any prior training in pedagogy (Balleux, 2011; Beaucher, 2021; Berger et al., 2018; Coulombe et al., 2020; Fejes & Köpsén, 2014). Different motivations for trade experts to become VET teachers have been described in the scientific literature, whether it is to improve their working conditions, to satisfy their desire to pass on their professional knowledge or simply to take on a new professional challenge (Balleux, 2006; Deschenaux et Roussel, 2008).

As they are not generally trained in pedagogy, new VET teachers must enroll in a mandatory teaching bachelor’s program once they are fully in charge of a class and its educational responsibilities. The 120-credit bachelor’s degree in vocational education aims to develop thirteen professional competencies prescribed by the Quebec education department (ministère de l’Éducation du Québec) such as “master the language of instruction”, “plan teaching and learning situation”, “evaluate learning, manage how the class operates”, “mobilize digital technologies”, “act in accordance with the ethical principles of the profession” (Gouvernement du Québec, 2020). Not surprisingly, “the bachelor’s degree followed by these teachers while starting the profession carries an extra burden in addition to the shock of entering a profession that is very different from the one they know” (Roussel, 2016, p. 145). Moreover, for many of them, the studies they have completed to obtain their professional trade qualifications (often a secondary-level Diploma of Vocational Studies) do not adequately prepare them for university-level training (Balleux, 2011; Roussel, 2016).

This atypical induction path creates not only significant stress for those who experience it but also does not encourage the appropriation of teaching practices and tasks (Balleux, 2011). Yet, as Beaucher (2010) points out, “it is on their shoulders that rests a large part of the heavy responsibility of offering quality training to students and ensuring the competence of Quebec’s future workforce” (p. 64). Thus, faced with the considerable changes they experience and the complexity of their new functions, these teachers are confronted with major challenges. Knowledge of curricula, instructional planning, representation of the teaching task as a whole, classroom management, and the heterogeneity of the student body are the main challenges facing VET teachers (Boldrini et al., 2018; Coulombe et al., 2010). As noted by Sarastuen (2020), although some new teachers access the opportunity to participate in coaching and training through apprenticeship, they nonetheless quickly realize that their skills and professional experiences are insufficient to fulfill their new professional role.

While many novice VET teachers will overcome the challenges associated with entering the profession, there are still significant attrition rates during the first few years of employment (Deever et al., 2020; Green, 2015; Tardif, 2001). As far back as 20 years ago, a study by Tardif (2001) revealed that 25% of VET Quebec teachers left the profession within their first year of teaching, and a third within the first five years. To our knowledge, no recent study provides a portrait of the current situation with regard to the professional dropout rates of new teachers. However, the scientific literature does indicate that the issues and challenges related to their professional induction have changed very little, suggesting that the reality is still worrisome (Beaucher, 2021; Beaucher et al., 2016; Coulombe et al., 2017; Roussel, 2016; Tremblay, 2020).

In light of the challenges faced by new VET teachers, it is essential to study the ways that facilitate their induction experience, accelerate the development of their professional teaching skills and increase their comfort level as they transition into their new career. The literature about early career teacher points to several factors that can facilitate induction into the profession, such as mentoring programs, early career training seminars, a collegial dynamic in the work environment and positive interaction with colleagues (Coppe et al., 2023; Gagnon, 2017; Thomas et al., 2020). Despite the fact that few studies have been conducted on support mechanisms for the development of these skills (Coulombe et al., 2016; Gagnon & Mazalon, 2016; Geeraerts et al., 2015), research on VET teachers remains silent on self-efficacy beliefs, an imperative aspect to study the vast field of human skills. The importance of teachers’ self-efficacy beliefs in practice is well established. Previous research has also shown that teachers’ self-efficacy beliefs differ according to their experience and the professional context in which they teach (see Gaudreau et al., 2012, for a review). Considering the unique situation of VET teachers, it is essential to examine the single case of VET teachers during the critical period of their professional induction.

## Theoretical Background: Self-efficacy Beliefs

The concept of self-efficacy was first introduced by Bandura (1977) as part of his social cognitive theory. This theory studies human phenomena through the interaction of three variables—behaviors, individual characteristics and environment—each of which is influenced by the other two (Gagnon, 2017). As suggested by its name, the theory prominently features cognitive factors. To the contrary of the behaviorist theories of learning that was popular at the time, Bandura asserted that humans do not simply respond to stimuli, but interpret them. Individual characteristic can thus influence both behavior and perceptions of the environment, and in this sense, everyone takes an active part in their own development. Therefore, people can demonstrate perseverance in their actions even if they do not receive immediate positive feedback from their environment or major external support. Bandura precisely introduced the concept of self-efficacy to explain this ability for individuals to encourage themselves and to persevere in their behaviors. Self-efficacy can be understood as an individual’s set of judgments and beliefs about his or her capacity to act in the ways necessary to reach specific goals (Bandura, 1977, 1986, 1997). In other words, self-efficacy is about an individual’s ability to organize his thoughts, actions and efforts, and deploy strategies to overcome challenges (Tschannen-Moran & Hoy, 2007).

For Bandura (1997), perceived self-efficacy is the most important determinant of human action: it influences how people feel, think, motivate themselves, and behave. It is “a key factor in a productive system of human competence” (Bandura, 2019, p. 73). Indeed, throughout their lives, the particular forms of competence that individuals acquire are the expression of natural gifts, sociocultural experiences, and fortuitous circumstances that alter their developmental trajectories (Bandura, 1986). These different formative influences shape an individual’s self-knowledge, and consequently, their beliefs about their competencies. By having an impact on control over action, self-regulation of cognitive processes, motivation, and emotional states, “perceived self-efficacy thus contributes strongly to performance, regardless of the skills involved” (Bandura, 2019, p. 73). Having strong self-efficacy beliefs will have multiple positive effects in an individual’s life, as pointed out by Lecompte (2004), who explains the mechanisms of action:

People who believe strongly in their capacities approach difficult tasks as challenges to be met rather than threats to be avoided, which increases their interest in them. They set challenging goals and maintain a high level of commitment to them, investing a lot of effort and increasing it in the event of failure or setback. They remain focused on the task and think strategically in the face of difficulties. These people ascribe failure to a lack of effort, which fosters a mindset geared towards success; they thus quickly recover their sense of efficacy after a failure or a decline in performance. Finally, they approach potential threats or stressors with the confidence that they can exercise some control over them. (p. 60)

During the 1980s, the concept of self-efficacy became increasingly popular, with more attention devoted to its possible applications in the teaching profession. In educational research, a keen interest was developed in “teacher self-efficacy” which is defined, according to Dellinger and his colleagues (2008), as the belief a teacher has in their ability to successfully perform specific teaching and learning tasks in the classroom. Studies have shown that teachers with higher levels of self-efficacy present their lessons more effectively, demonstrate better classroom management, and interact with students in ways that are more responsive to their needs (Tschannen-Moran & Hoy, 2001). Positive correlations have also been established between perceived self-efficacy and student engagement, motivation, and academic achievements (Abu-Tineh et al., 2011; Aldridge & Fraser, 2016; Gaudreau, 2017; Guo et al., 2012; Klassen & Tze, 2014). Furthermore, teachers with strong self-efficacy beliefs not only perceive their profession as less stressful than do their colleagues with a low sense of efficacy (Klassen & Chiu, 2010), but also appear to be less affected by work absenteeism (Brouwers & Tomic, 2000). A strong sense of self-efficacy also seems to be associated with a higher level of professional engagement and motivation, as well as being a favorable factor in preventing burnouts, and potentially higher retention opportunities in the profession (Hultell et al., 2013; Schwarzer & Hallum, 2008).

There is an abundance of research that demonstrates the positive impacts of strong self-efficacy beliefs on various aspects of teaching. However, very few studies have examined VET teachers’ self-efficacy beliefs during the critical period of their induction into the profession. Although perceived self-efficacy is far from being the only factor determining the motivation, commitment and performance of new teachers, it is nevertheless imperative to explore how to support them in their professional development in order to better act as such. To do so, supporting the development of a proactive attitude among new teachers can be done by focusing on what they can control. Consequently, the purpose of this article is to answer the following question: what strategies do new VET teachers used to increase and maintain their sense of efficacy?

Wishing to fill some of the gaps in the scientific literature, the value of this research also rests on the mechanisms by which self-efficacy beliefs operate. Indeed, in order to have an impact on self-efficacy, an experience must first be cognitively processed: it is not the experience itself that impacts the individual’s self-efficacy, but rather the fact of becoming aware of it, reflecting on it, and evaluating its importance for oneself:

Information relevant to assessing personal capabilities [...] is not enlightening in itself. It only becomes instructive through cognitive processing of information on efficiency and through reflective thinking (Bandura, 2019, p. 134).

Therefore, by inviting participants to engage in such a cognitive process, it may improve their perception of effectiveness and hopefully, facilitate their professional induction.

## Methodological Framework

The purpose of this study was to understand the process by which new VET teachers develop and maintain their self-efficacy beliefs. This paper focuses on results related to one of the two research sub-questions concerning the strategies mobilized to increase and maintain efficacy beliefs. Because the study was set in an interactive dynamic between the researcher and participants, in a context tinged by their representations and personal experiences, an interpretative posture was adopted and, consequently, qualitative methods of data collection and analysis were favored (Savoie-Zajc, 2011).

### Study Specifications

Participants were recruited through an email invitation sent to the administration of 40 VTC in Quebec (Canada). These VTCs were selected in order to maximize representation of the various sectors of vocational training and to encourage the participation of teachers from institutions of various sizes and locations. An administrator at each location was asked to contact all their teaching staff with an invitation to participate in the research. This invitation stated the objective of the research (gathering the perceptions of individuals in the induction phase of the teaching profession) and the criteria for study participation, including that participants must be teachers employed by a VTC, and had to be for less than five years.

A total of 21 teachers (11 men and 10 women) from 8 of the 21 vocational education sectors, expressed interest in participating in the study (Administration, Commerce and Computer Technology, Beauty Care, Environment and Forest Management, Forestry and Pulp and Paper, Health Services, Metallurgical Technology, Mining and Site Operation, Motorized Equipment Maintenance). The profile of participants is detailed in Table 1.

**Table 1:** Vocational training programs taught by the participants, years of experience in their trade and years of experience in teaching

Participants (pseudonyms)	Vocational training programs taught	Age	Years of experience in their trade	Years of experience in teaching
*Alexandre*	Heavy-duty road vehicle mechanics	36	14	4
*Claude*	Construction equipment mechanics	38	3	2
*Florence*	Starting a business	40	15	5
*Frédérique*	Protection and development of wildlife habitats	45	6	4
*Henry*	Construction equipment operation	46	10	2
*Jean*	IT support	38	10	1
*Johanne*	Forest management + Protection and development of wildlife habitats	32	7	3
*Juliette*	Aesthetics + Hair removal	34	14	2
*Louisa*	Accounting + Secretarial studies	40	11	1
*Lucas*	Welding-assembly	41	14	1
*Marc-Antoine*	Heavy-duty road vehicle mechanics	37	6	3
*Malik*	Construction equipment operation	44	21	4
*Marianne*	Starting a business	45	10	2
*Maryse*	Accounting + Secretarial studies + Starting a business	32	9	1
*Mila*	Pharmacy technical assistance	37	12	2
*Nadège*	Accounting + Secretarial studies	33	10	2
*Natasha*	Accounting + Secretarial studies + Secretarial studies—medical	33	11	4
*Normand*	Construction equipment mechanics	34	25	3
*Patrice*	Welding-assembly	37	14	5
*Pierre-Olivier*	Agricultural mechanics	27	10	2
*Yves*	Heavy-duty road vehicle mechanics	36	4	2

At the time of data collection (fall 2019 and winter 2020), participants ranged in age from 27 to 46 years (m = 37) and had an average of 2.6 years of teaching experience. Twelve participants had earned a Diploma of Vocational Studies as their highest degree, five held a Diploma of College Studies, one held an undergraduate diploma, and three held a graduate diploma (master). In addition, at the time of the interview, all participants were enrolled in a bachelor’s degree program in vocational education.

### Data Collection and Analysis

Data were collected through a 75–90 min semi-structured individual interview (Savoie-Zajc, 2009). The interviews were conducted by the main researcher helped by two research assistants. The questions asked focused on: the participants’ overall induction experience, their perceived self-efficacy as teachers, strategies they used to develop and maintain their self-efficacy during this critical period (e.g., “Do you feel effective in your work in general and can you explain where this feeling comes from?”; “What do you do, concretely, to feel more effective in your work?”; “If we take the example of the situation that we talked about in which you felt ineffective; how would you make it possible for you to feel more effective if this situation presented itself again?”).

Recorded interviews were transcribed and the data were imported and processed using the software *NVivo* *12*. A semi-inductive logic characterized the data analysis where the researcher would question the meaning contained in the collected data while allowing the key research concept (self-efficacy) to guide the analysis. This flexible procedure allowed the researchers and the assistants to constantly adjust the data classification during the process of analysis (Savoie-Zajc, 2011). A detailed line-by-line analysis of each interview was then undertaken. This is what Strauss and Corbin (2004) call microanalysis, which involves “examining and interpreting the data very carefully, even meticulously” (p. 84). According to these authors, microanalysing involves dissecting the data, paragraphs, sentences and words contained in the interviews in order to identify the general ideas that emanate from the participant’s words and to grasp their meaning. To do so, Strauss and Corbin (2004) suggest asking the following questions while reading every sentence of an interview: “How do we interpret what the interviewee is saying?” and “What is in this material?” (p. 85). These questions were after being used to assign codes from the full transcripts of the interviews and to identify and describe the first categories (Blais & Martineau, 2006). Seventy-seven codes were thus created and classified into five categories, i.e., they were grouped according to their proximity of meaning and similarities (Strauss and Corbin, 2004). Of those seventy-seven codes, twenty-three were about strategies to increase and maintain self-efficacy (e.g., STRATEGY-student relations, STRATEGY-formative assessment, STRATEGY-Sport and leisure), which were then grouped into the four categories presented in this article.

Blind parallel coding procedures were also applied to ensure the rigor of analysis. Firstly, the main researcher conducted an analysis of three of the twenty-one interviews and developed a first set of codes. A second researcher proceeded to analyze these three same interviews, creating a new list of codes. To ensure a common understanding of the meaning given to the data, these two lists were then compared, reorganized partially, and redescribed, if necessary. The remaining interviews were then analyzed with this last list, while allowing the addition of new codes if necessary.

## Results: Strategies used by VET teachers to improve their self-efficacy

Following analysis of the interviews, twenty-three strategies for developing and maintaining self-efficacy were identified. As announced, these strategies were grouped into four main categories: 1) *strategies related to the work of teaching;* 2) *strategies related to the mobilization of human resources*; 3) *strategies related to professional development;* and 4) *strategies related to attitudes and well-being*. The following sections describe each of these categories, alongside with their corresponding strategies, using excerpts from the collected testimonies (Table 2). Pseudonyms were used to ensure the anonymity of participants.

**Table 2:** Strategies used by VET teachers to improve their self-efficacy

**Strategies related to the work of teaching**	1. Having good teaching preparation and planning 2. Building and maintaining a good relationship with the students 3. Using frequent formative assessments 4. Using good quality didactic materials
**Strategies related to the mobilization of human resources**	1. Seeking help and support from fellow teachers 2. Calling on the various resource persons available at the VTC 3. Observing experienced colleagues and taking advantage of their wide knowledge 4. Mobilizing their former professional network
**Strategies related to professional development**	1. Doing formal and independent training 2. Assessing and learning from their experiences 3. Being part of strategic groups
**Strategies related to attitudes and well-being**	1. Approaching tasks with positivity 2. Getting involved in the tasks and life of the VTC 3. Practicing a sport or hobby that we enjoy

### Strategies Related to the Work of Teaching

Some strategies identified by the participants as maintaining and developing their self-efficacy refer directly to their work as teachers. These actions (initiatives, interventions, habits) were implemented in context, and concern didactic and pedagogical aspects of their work. In this matter, for nearly all participants, self-efficacy was closely correlated to *teaching preparation and planning*. Indeed, several teachers mention that they invest a lot of energy in planning their teaching. Alexandre, for example, emphasizes the importance of referring to the curriculum and taking the time to critically analyze it in order to determine the elements to teach and the essential knowledge to address in his lectures:

*I’ll scrutinize the program to see where I’m at: ‘That, I’ve covered. And this, is that really important? Is that where we’re at in the industry?’—I’ll analyze what I’m going to teach them. It has to be really useful to them.*

For some, this preparation allows them to plan their interactions with students more effectively, to anticipate difficulties or unforeseen events, as well as to be prepared with solutions, allowing them to considerably increase their teaching productivity. For others, it is a way to ensure fluidity and continuity in the teaching–learning process: “*Planning is really the key. Since I am super-organized and structured, I know exactly where my students are at; which module, when they are going to be ready for the exam. I’m able to know several weeks in advance* […]” (Nadège).

For Lucas, as for nine other participants, *building and maintaining a good relationship with their students* is the strategy that allows them to feel effective. Getting to know their students makes them feel better equipped to guide them, to understand their challenges, and to provide the help and support they need. To be able to gain the trust of students is also interpreted by many as a sign of efficacy.

The *frequent use of formative assessments* was also identified by a quarter of the participants as a winning strategy. In addition to validating the efficacy of the teaching and learning methods used, formative assessments provide crucial information about students’ needs, thereby, guiding future interventions and facilitating future lesson planning.

Lastly, *the didactic materials used* can also have an impact on perceived self-efficacy. Having recent or up-to-date material that reflects the reality of the labor market allows some teachers to feel more effective in their teaching. For Pierre-Olivier, who teaches construction equipment mechanics, this is reflected in the use of laptops during practical workshop (e.g., in the VTC garage). This mobilization of digital resources as part of his teaching allows him to feel equipped to then better feed his students, who will be called upon to use these resources once they enter the workforce. Moving from paper documents and technical manuals to computers also makes him more confident, he explains, because “[…] *when someone asks a question, you don’t always have the answer* […] *but you have all the information on the computer*” (Pierre-Olivier).

### Strategies Related to the Mobilization of Human Resources

Several strategies for developing and maintaining self-efficacy concern the ability to successfully mobilize the resources available in one’s environment. In fact, for the majority of participants, *seeking help and support from fellow teachers* is a strategy that had considerable implication on their self-efficacy beliefs. Ten participants mention that they had sought out one or more colleagues for advice on various aspects of their teaching practice. Louisa explains how her experienced colleague helped her feel more comfortable with the curriculum: “*The person I work with, she has twenty years of experience in vocational education, so often I’ll draw on her experience and benefit from her wide knowledge of the curriculum*”. Others, like Marianne, seek the opportunity to observe colleagues’ teaching and to solicit their feedback: “*Out of concern for doing well, I invited my colleague, so she observed my classes and gave me advice on how to organize myself better and also on how to enliven the lecture*”. For some, the support of colleagues was crucial, to the point where the mutual aid in their environment was a deal breaker; they would probably have left the profession without it. In sum, it is through such exchanges, discussions, and mutual support between colleagues, through the mobilization of their knowledge, know-how, and interpersonal skills, that new teachers find solutions to the challenges they face: “*I often say: ‘Individually, we are worth nothing and collectively, we’ll get by’*” (Patrice).

Just over half of the participants mention the importance of *calling on the various resource persons available at the VTC*, whether that was school support staff, an education consultant, a member of management or a social worker. Like many of her colleagues, Maryse recognizes the importance of orienting her students to specialized resources when facing issues for which she does not feel equipped (e.g., emotionally charged situations): “*I think it’s about referring them to the right resources, reframing my role, my functions. I can say to the student, ‘Yes, I hear what you’re saying, I’m listening, however, I’m going to refer you to that person’—something I’m doing a little bit more now*”. Most of the time, participants report asking help from a resource person to intervene with students who are experiencing, for example, learning difficulties, substance abuse problems, family or financial difficulties.

Another way to mobilize resources in the environment is to *observe experienced colleagues and take advantage of their wide knowledge*, to draw inspiration from their daily practices such as remedial activities. Nadège, an accounting teacher, explains how observing her colleague inspires her own practice in terms of how to interact with students who have more difficulty achieving course goals: [...] *she does remedial workshops, for example, on debit/credit. And, even though it’s a concept for me that’s acquired, I go to her remedial workshops as an observer to see how she interacts with students, and what kinds of questions she asks*.

Juliette, for whom the observation of colleagues has been the most helpful, even says that this strategy should be mandatory for all new teachers in order to increase their self-efficacy.

Lastly, some teachers mention that the *mobilization of their former professional network* allows them to feel effective by benefiting from the occasional support of “experts in the field”. For example, when the new teachers have interrogations about specific equipment used in the industry or standards in practice settings, they will reach out to their former colleagues in the workforce to validate technical or practical aspects.

### Strategies Related to Professional Development

In order to increase their self-efficacy, the teachers interviewed also highlight the importance of professional development, both at the pedagogical level and in respect to their field of expertise. Thus, for half of the participants, engaging in *formal and independent training* is a strategy to remain connected and stay up to date in the field of the trade they teach:

[...] *to feel effective in my work, I try to keep myself up to date.* [...] *It’s registering for small training workshops, it’s talking with employers, it’s understanding what’s new in the labor market, it’s trying to keep up with technology* [...] (Natasha).

Florence says that training and independent study allowed her to increase her repertoire of actions, and indirectly, the number of students enrolled in her program: “*I read a lot, I keep up to date, I go to a lot of conferences, I go outside of my Center a lot* […].”

Coherently, six of the teachers mention the importance of *assessing and learning from their experiences*. For Juliette, this takes the form of weekly check-ins where she analyzes the different tasks that made up her work week. As the teaching days go by quickly, it is easy, she explains, to forget everything that has been accomplished and to move on to the following week, without recognizing the importance of the work carried out and/or learning from one’s experiences. For Johanne, the quality of her teaching relies, among other things, on her ability to reflect on her previous planning, and to constantly readjust it:

*I’m a list person.* [...] *last June, there was the Animal Pathology course that I wanted to improve. Well, I opened my agenda for the past year: ‘What did I manage to do?’ I had added boxes to my list. And then, I had checked them off. I had planned to do three things, but I just did two. ‘When had I done the pretest?’ I had put a note in my calendar: the students thought it was too early*.

Finally, some participants identify as *being part of strategic groups* as a winning strategy for developing and maintaining their self-efficacy in teaching. New teachers explain that this strategy (being a member of a company’s board of directors or a trade association) allows them to keep a foot “in the field”. In addition to staying informed about the changes and developments in their field of expertise, teachers say this also allows them to provide better service to their students, including ensuring a higher quality of training and connecting potential employers with graduating students.

### Strategies Related to Attitude and Well-being

In an effort to feel professionally effective, several teachers indicate that they strive to adopt positive attitudes and behaviors related to their well-being. First, nearly half of the participants express the importance of *approaching tasks with positivity*. For some, this means consciously choosing to focus on the positive aspects of their work even in more difficult times. For others, it means putting in place, at key moments in their journey, a certain attitude of “letting go”, allowing them to focus their energies on what really matters, and thus, avoid burnout. For Natasha, who was negatively affected by certain difficult relationships with students when she first started her teaching journey, this meant to ground her sense of self-efficacy on her best practices. Therefore, she remembers a saying from her school principal that contributed to change her perception and to approach her job with more positivity:

*I’ve been told over and over again not to take it personally. Then my manager said to me, ‘Are you going to base your confidence, your effectiveness, on a situation like this versus all the other positive ones?’ No!*

*Getting involved in the tasks and life of their VTC* is also a strategy reported by participants that allows them to feel useful, valued, and important. For instance, involvement in VTC committees or promotional activities represent an example of their effectiveness in the profession.

For a quarter of the participants, effectiveness at work does not depend solely on their well-being within the professional sphere. Like Frédérique, several of the teachers mention that *practicing a sport or hobby* they particularly enjoy can have a positive impact on their effectiveness at work: “*I’m currently doing yoga (laughs). It sounds silly, but it calms me down a lot. Feeling calm allows me to better discuss with my colleagues, to be much calmer, and to be receptive as well”.* In the same way, sports and leisure time allow some new teachers to maintain an adequate energy level for teaching and a balance between their personal and professional lives.

## Discussion

This study has identified a range of strategies that seems to have contributed to maintain and increase VET teachers’ self-efficacy beliefs. Some observations can be made about the different categories of strategies used.

*Strategies related to the work of teaching* were the ones most frequently reported by participants. These strategies include several elements of the teaching context such as the curriculum planning process, the knowledge of the curriculum, the relationships with students, self-evaluation to support learning, the assessment of the relevance of contents and teaching materials. That being said, unanimously, the participants believed that planning teaching activities was the best strategy for maintaining and increasing perceived self-efficacy. With the goals of structuring and coordinating a series of activities into a coherent lesson that promotes meaningful student learning, instructional planning is central to the act of teaching and closely associated with the quality of the teaching itself (John, 2006; Ruys et al., 2012). Its importance is notably explained by its highly reflective nature and its impact on several components of the teaching–learning situation, as well as on the quality of student learning. In fact, instructional planning affects many aspects, such as the choice of contents, the time devoted to them, the teaching, the learning, and the evaluation strategies employed, the teaching materials used, and the means of differentiating instruction. Some authors have suggested that this is the most important skill in the teaching–learning process (Gagné & Berger, 2019). Others have suggested that the quality of instructional planning improves the quality of delivery (Dorovolomo et al., 2010) and therefore, that instructional planning indirectly improves the quality of student learning (Mirza et al., 2022; Poulin et al., 2015). While the present study does not allow us to evaluate the quality of the participants’ actual skills in instructional planning, it does enable us to conclude that novice teachers are aware of the great importance of instructional planning in the teaching–learning process. Furthermore, as instructional planning has been identified as one of the main challenges for new teachers in the vocational sector (Coulombe et al., 2010), this skill should be one of the first to be addressed in VET teacher training, whether at the university level, in support programs for professional induction, or in ad hoc training offered by schools.

*Strategies related to the mobilization of human resources* are also highly valued by teachers to maintain and increase their sense of self-efficacy. Many of the teachers relied on their colleagues and the various resource people at their VTC to increase their belief in their teaching effectiveness. It is not surprising that the support of various players in the school environment plays a substantial role in this increase. Indeed, being in the early stages of their university training and teaching experiences means that novice teachers recognize that the help and the collaboration of their school staff team are essential to successfully accomplishing their new teaching tasks. In 2008, as part of a day of study on the theme of the success and perseverance of vocational training students, participating VET teachers expressed significant needs for support and the help of specialists to adequately perform the tasks assigned to them (Beaucher et al., 2016). Our results are consistent with the literature on professional induction, which advocates for teacher networking so that experienced teachers can become mentors to novice teachers in their schools (Carpentier et al., 2020).

Regarding *strategies related to professional development*, of the 10 participants who listed formal and independent training as a strategy for increasing their self-efficacy, only 3 referred to pedagogical development. Of these same participants, 9 reported ways in which they contributed to increase their self-efficacy through training or self-training in relation to content taught in their curriculum. It seems, therefore, that the perceived effectiveness of VET teachers is related more to their knowledge of the field, know-how, and interpersonal skills (i.e., a thorough understanding of the subject being taught) than to their pedagogical knowledge (i.e., developing a quality relationship with students, varying their teaching methods). In this manner, the damage to have significant challenges in their trade content knowledge (being recognized as experts in their field) would be greater in regards of their professional self and identity; it would be more acceptable for them to have gaps in their pedagogical knowledge (being novices in teaching). This finding also echoes studies that have examined the professional identity of VET teachers. As mentioned earlier, when new teachers enter the profession, they undergo a major identity transition: while they are “no longer entirely workers in their profession, they are not entirely teachers in a training center yet” (Balleux, 2011, p. 33). As they struggle to define their professional status during this period of induction, it is their sense of belonging to their previous profession that is strongest, rather than their sense of belonging in the teaching field (Deschenaux & Roussel, 2008). It is therefore not surprising to find that the professional development strategies mobilized to maintain and increase self-efficacy are associated more with their identity as a tradesperson than as a teacher. The implementation of a mentoring system (experienced VET teachers supporting new ones) could serve to support VET teachers in this professional identity transition and help them maintain and increase their self-efficacy.

For many participants, the positive ways in which they choose to approach either their day-to-day work or their respond to challenging events related to their teaching practice appear to contribute significantly to their beliefs of self-efficacy in teaching. This finding can be interpreted in light of cognitive psychology. The “science of positive subjective experience, positive individual traits, and positive institutions” (Seligman & Csikszentmihalyi, 2000, p. 5)—a theory whose relevance as the basis of interventions to promote well-being and alleviate depressive symptoms—has been empirically demonstrated in a variety of settings (e.g., work, school, psychological counselling clinics) and for various types of individuals (e.g., teachers, students, patients, caregivers; see, e.g., meta-analyses by Bolier et al., 2013, and Sin & Lyubomirsky, 2009). However, such devices, including the use of strategies to encourage novice teachers to approach tasks with positivism, have not been included and routinely offered to teachers during the induction period.

Ultimately, in order to have an impact on perceived self-efficacy, an experience must first be cognitively processed by an individual. Thus, much like the participants were asked to do during the research interviews, it is by becoming aware of it, reflecting on it, and evaluating its importance for themselves that the experience will have an impact on self-efficacy beliefs. It seems important, therefore, that induction support systems have strategies and mechanisms in place to encourage reflection on the different experiences teachers can draw upon to build their effectiveness and the types of strategies that can be mobilized to do so. Moreover, these mechanisms should support and encourage novice teachers to approach their professional development and learning of new teaching tasks with positivism (Donaldson et al., 2019; Vaart et al., 2019). In addition, these support systems should include activities to educate new teachers about the importance of a strong sense of efficacy linked to a successful induction into the profession. These workshops should recall the mechanisms by which efficacy beliefs operate, as well as the role of positive beliefs in the development of the latter and in the quality of an individual’s achievements. In this sense, trainers and mentors who work with these novice teachers should be focused, particularly through reflective practice (Lefebvre & Thibodeau, 2015), on fostering awareness among their protégés regarding the various ways, both in their personal and professional lives, to develop their self-efficacy beliefs as teachers.

## Limitations and future directions

This study has some limitations that should be reported and considered in future research. First, this study only concerns the participants’ perceptions of effectiveness and therefore, their self-efficacy level was not measured, in any way. Therefore, the tangible impacts of the strategies mentioned by the participants on their efficacy beliefs cannot be clearly assessed and remain within the realm of perception. Also, the results are based on a small convenience sample, which limits the possibility of generalizing these results to the entire VET teaching population. Therefore, it would be desirable to replicate the results of this study using other samples (including schools of different sizes and regions) in order to increase the richness of the strategies that novice teachers believe helpful during the induction period. Moreover, the use of a larger sample would make it possible to consider the potential influence of different variables such as the composition of the groups of students, the training program taught, or the educational context advocated in the school (e.g., group or individual teaching). We believe this would contribute to enriching the understanding of the unique reality of these novice teachers. Another limitation is the exclusive use of interviews to investigate the topic under study. This may have increased the risk of social desirability and influenced the results obtained. To overcome this limitation, it would be helpful to replicate the results of this study using another method to collect data such as observations in the classroom and in the work environment. In future research, it would be relevant to take into account the motivators to become a VET teacher and if or how these motivators could have an influence on a) the strategies deployed and b) on self-efficacy beliefs.

The literature shows a variety of factors that can influence the quality of teachers’ professional induction such as mentoring, induction activities and kits, in service training and seminars, classroom observation of an experienced teacher, joint planning time or support groups (Gagnon 2017; Mukamurera et al., 2019; Ronfeldt & McQueen, 2017). It has also been documented that a collegiate and collaborative school culture including caring professional network can also greatly facilitate the induction of novice teachers (Coppe et al., 2023; Thomas et al., 2020). Consequently, the school’s administrators and pedagogical leaders have a decisive role to play in creating the right conditions for novice teachers to succeed in their first years of teaching (Deever et al., 2020; Gagnon, 2017). It is the presence of a multitude of these factors that will make a real difference in how the early career years are experienced. It is also worth mentioning that, each in their own way, these conditions can have a positive impact on the recruits’ self-efficacy beliefs. Knowing which strategies teachers use to develop their self-efficacy is one of the many levers on wich it is possible to act. However, we believe the “smallest” factor is and can be relevant to analyze the influence of any other factors, or all of them, over which teachers have some control such as strategies for developing self-efficacy beliefs. In this sense, the present study is innovative in proposing a study of 21 new VET teachers and identifying the different types of strategies supporting the development and maintenance of their perceived self-efficacy. The identification of these strategies allowed for a deeper understanding of previously unexplored aspects of the realities of these teachers. In addition, the new knowledge acquired could lead to the improvement of the induction experience of VET teachers by offering avenues for developing interventions targeted to the needs of novice teachers.

In a context where the issue of professional induction is of concern to many educational researchers and where there is a growing emphasis on ways to optimize the skills and professional practices of new teachers, studies of their self-efficacy beliefs are most relevant. This paper aims to facilitate the elaboration of training programs and professional development within VTCs and hoped that these findings will encourage new teachers to be proactive in identifying ways in which they can develop their self-efficacy, and thereby positively impact their professional practice. A deeper understanding of how teachers optimize their perceived self-efficacy may lead to better work motivation, reduced risk of dropping out of the profession, and consequently, positive effects on the quality of education provided to students in the various vocational training sectors in Quebec, and hopefully, elsewhere.

These findings also lead to recommend that the bachelor’s degrees in vocational education should include to the training the objective of providing to the new teachers the necessary support to enable them to be quickly equipped in terms of planning teaching and learning situation. This university training should include more time to the well-being in the teaching profession and to the promotion of the different strategies to cultivate it on a daily basis. Finally, this program should support, accompany, and nurture the ability of new VET teachers to develop their self-efficacy beliefs by becoming aware of their past experiences, reflecting on it, and evaluating their importance for themselves and their teaching career.

## Data Availability

The datasets generated during and analysed during the current study are available from the corresponding author on reasonable request.

## Footnotes

1 Vocational training programs correspond to Level 3 of the International Standard Classification of Education (ISCED). “Training programs in level 3 are conceived to prepare for higher education, and/or to teach skills relevant to employment” (e.g., plumbing and other trades).

2 A Vocational training centre is an educational institution where one or more vocational training programs are taught.
